# Mesenchymal stem cells and their extracellular vesicle therapy for neurological disorders: traumatic brain injury and beyond

**DOI:** 10.3389/fneur.2025.1472679

**Published:** 2025-02-05

**Authors:** Aref Yarahmadi, Masoumeh Dorri Giv, Reza Hosseininejad, Azin Rezaie, Narges Mohammadi, Hamed Afkhami, Arastoo Farokhi

**Affiliations:** ^1^Department of Biology, Khorramabad Branch, Islamic Azad University, Khorramabad, Iran; ^2^Nuclear Medicine Research Center, Ghaem Hospital, Mashhad University of Medical Sciences, Mashhad, Iran; ^3^Department of Stem Cells and Developmental Biology, Cell Science Research Center, Royan Institute for Stem Cell Biology and Technology, ACECR, Tehran, Iran; ^4^Department of Microbiology, Faculty of Biological Sciences, North Tehran Branch, Islamic Azad University, Tehran, Iran; ^5^Department of Molecular Cell Biology and Microbiology, Faculty of Biological Sciences and Technologies, University of Isfahan, Isfahan, Iran; ^6^Cellular and Molecular Research Center, Qom University of Medical Sciences, Qom, Iran; ^7^Nervous System Stem Cells Research Center, Semnan University of Medical Sciences, Semnan, Iran; ^8^Department of Medical Microbiology, Faculty of Medicine, Shahed University, Tehran, Iran; ^9^Department of Anesthesiology, Kermanshah University of Medical Sciences, Imam Reza Hospital, Kermanshah, Iran

**Keywords:** brain, traumatic brain injury (TBI), mesenchymal stem cells (MSCs), cell therapy, extracellular vesicles (EVs)

## Abstract

Traumatic brain injury (TBI) is a complex condition involving mechanisms that lead to brain dysfunction and nerve damage, resulting in significant morbidity and mortality globally. Affecting ~50 million people annually, TBI's impact includes a high death rate, exceeding that of heart disease and cancer. Complications arising from TBI encompass concussion, cerebral hemorrhage, tumors, encephalitis, delayed apoptosis, and necrosis. Current treatment methods, such as pharmacotherapy with dihydropyridines, high-pressure oxygen therapy, behavioral therapy, and non-invasive brain stimulation, have shown limited efficacy. A comprehensive understanding of vascular components is essential for developing new treatments to improve blood vessel-related brain damage. Recently, mesenchymal stem cells (MSCs) have shown promising results in repairing and mitigating brain damage. Studies indicate that MSCs can promote neurogenesis and angiogenesis through various mechanisms, including releasing bioactive molecules and extracellular vesicles (EVs), which help reduce neuroinflammation. In research, the distinctive characteristics of MSCs have positioned them as highly desirable cell sources. Extensive investigations have been conducted on the regulatory properties of MSCs and their manipulation, tagging, and transportation techniques for brain-related applications. This review explores the progress and prospects of MSC therapy in TBI, focusing on mechanisms of action, therapeutic benefits, and the challenges and potential limitations of using MSCs in treating neurological disorders.

## 1 Introduction

Traumatic brain injury (TBI) is a disruption in normal brain function caused by an external force, leading to various degrees of neuronal damage and brain dysfunction (1–3). TBI is the most common cause of death and disability in people under 40 years of age in the United Kingdom and the third leading cause of death worldwide (1, 4). The mortality rate is 3.5 times higher than that of heart disease and cancer in industrialized countries (5). TBI involves both primary and secondary injuries. Mechanical trauma initiates primary damage, which subsequently triggers a cascade of pathological changes, leading to secondary injury (6). TBI contributes to various complications, such as cerebral hemorrhage and concussion. Additionally, TBI-related processes can lead to delayed apoptosis and necrosis, affecting autophagy and neuronal count (5, 7). Intracerebral hemorrhage (ICH), referred to as cerebral bleeding, intraparenchymal bleeding, or hemorrhagic stroke, is sudden bleeding into the tissues of the mind or bleeding the mind tissue into its ventricles, or both, that can cause brain harm and be life-threatening (8). Bleeding happens in 46% of all TBIs and is progressively predominant in moderate and severe injuries. Brain damage brings about hemorrhage that can steadily advance over the initial 24–48 h. Sometimes, the spilling blood gathers outside the vessel, forming a hematoma (9, 10). One less investigated potential objective has been mined vasculature and its effect on TBI results. A significant result of TBI is immediate harm to the cerebral vasculature. We still need a complete understanding of vascular components that could improve new vascular therapies for TBI and vascular-related brain injuries (11).

**Figure 1:**
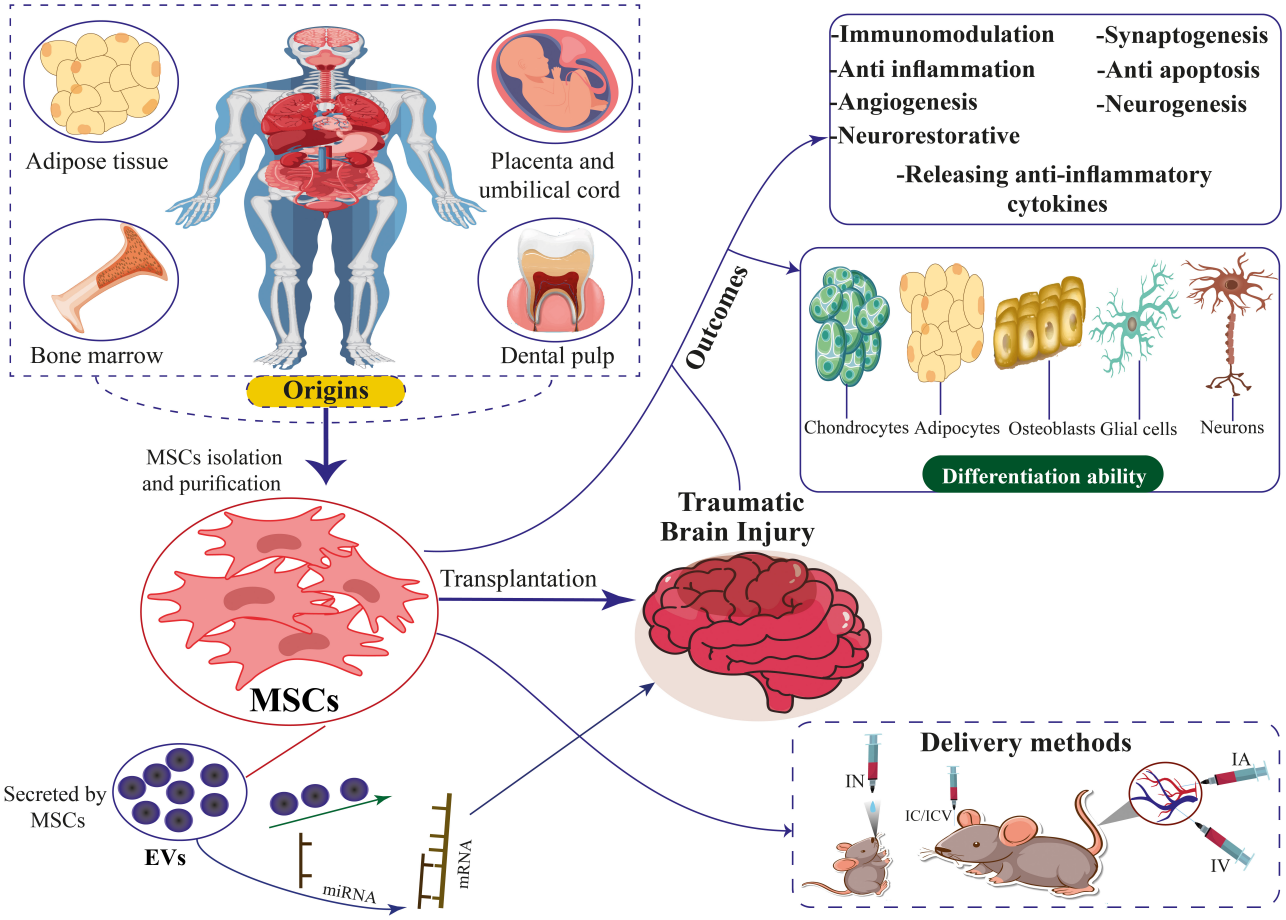
The origins, differentiation ability, therapeutic mechanisms, and delivery methods of MSCs and EVs secreted by MSCs for TBI. MSCs can differentiate into chondrocytes, adipocytes, neurons, osteoblasts, and glial cells.

At present, there is no safe drug treatment for moderate and severe TBI (12, 13). Among the pharmacological agents evaluated, nimodipine and nifedipine, both dihydropyridines, demonstrated efficacy in rodent and mammalian models. However, these agents showed limited effectiveness in promoting recovery in patients with TBI (14, 15). So far, high-pressure oxygen therapy, non-invasive brain stimulation, drug therapy, and behavioral therapy have been used to treat TBI. Recent advances in stem cell research have opened new avenues for treating TBI (16, 17). In recent years, progress in the study of stem cell biology, especially mesenchymal stem cells (MSCs), has been very effective in improving and repairing brain injuries, such as TBI, and therapeutic strategies for stroke treatment (Table 1) (6, 18). These cells can, by migrating to that area and replacing the damaged tissue or by differentiating into cells that secrete growth factors and anti-inflammatory cytokines, in addition to decreasing brain edema and neuroinflammation and, on the other hand, improving function. Movement increases the proliferation and differentiation of neural stem cells, neurogenesis, and angiogenesis (16, 19). Exosomes play a role in the recovery of perinatal brain damage and reduce neuroinflammation caused by microglia (20, 21). Based on studies, MSCs produce extracellular vesicles (EVs) that prevent microglia activation, and then exosomes are used to reduce neuroinflammation caused by TBI (Figure 1) (22, 23).

**Table 1:** The therapeutic effects of MSCs in all types of brain injuries.

**Type of brain injury**	**The therapeutic effect of MSC in all kinds of brain injuries**	**Route**	**References**
Ischemic stroke (ICH)	Nerve damage recovery by regulating various mechanisms, such as immune system function, nutritional factor secretion, stimulating angiogenesis, neurogenesis, and synapse formation.	IV, IA	(271–273)
Hemorrhage	Reduces brain damage after ICH through the Rhino signaling pathway and promotes neurogenesis.	Intraventricular, IV	(274–276)
Concussion	It improves neurological characteristics by differentiating the damaged areas of the brain into healthy neurons and increasing glial cells on the damaged site.	IV, IA	(208, 277)
Tumor	Apoptosis inducement through the phosphatidyl-3-kinase/protein kinase B (AKT/PI3K) pathway and growth suppression and proliferation of glioma cells and exhibition of anti-angiogenic characteristics.	IV, localized injection	(74, 278)
Encephalitis	In addition to regulating inflammation, the ability to migrate to the site of injury and differentiate into different types of cells, such as fat cells, osteocytes, chondrocytes, and neurons, causes a defensive immune function against many injuries caused by bacteria or viruses.	IV, intranasal administration, intraperitoneal	(250, 279)

IV, intravenous; IA, intra-arterial.

This article aims to investigate the familiarity with MSCs and their types, EVs derived from MSCs, the regenerative and therapeutic role of MSCs and EVs in TBI, and the challenges and potential limitations of MSCs used in treating neurological disorders.

## 2 Mesenchymal stem cells

MSCs are multipotent cells that can self-renew and differentiate into various cell types. They can be sourced from multiple human tissues and organs, including bone marrow (BM), adipose tissue, lung, brain, synovial fusion, pancreas, synovium, and peripheral blood (24–30). Friedenstein was the first to develop guinea pig bone-forming cells, and Owen revitalized this research by extending this technique to rats (31, 32). The isolation and *in vitro* expansion of MSCs derived from human BM were first documented in 1992, with subsequent reports of their therapeutic administration to patients commencing as early as 1993, as noted in a publication from 1995 (33, 34). Stem cells are characterized primarily by their multi-differentiation and self-renewal capabilities, as well as their various origins. Additionally, MSCs aid in tissue regeneration by producing cytokines and growth factors that draw other cells to the injured area (35–37). These cytokines and growth factors also encourage the development of new blood vessels, which are essential for tissue healing. Stem cell treatment is a viable alternative for tissue regeneration and repair since MSCs can control immune system activity, lower inflammation, and inhibit immunological responses. They are therefore excellent candidates for cellular treatments for a range of illnesses because of this characteristic (38–40). Stem cell transplantation has been shown in numerous studies to be beneficial for several diseases, including diabetic foot ulcers, congenital cataracts, ocular surface burns, severe skin burns, myocardial infarction, Parkinson's disease, Huntington's disease, and TBI (41–50). MSCs derived from human or rat donors specifically target damaged brain tissue (homing) following injections and aid in functional recovery (51, 52).

MSCs demonstrate therapeutic benefits in various medical conditions due to their diverse mechanisms of action. These cells are recognized for their regenerative properties, which encompass facilitating tissue regeneration and enhancing the healing process of wounds (45, 53, 54). The functions of MSCs in the management of neurological disorders and diseases encompass mitigating inflammation via immunomodulation, discharging trophic factors to facilitate therapeutic outcomes, fostering neurogenesis, being antibacterial, stimulating angiogenesis, diminishing infarct volume, substituting damaged cells, and emitting EVs, all of which contribute to their therapeutic efficacy (55–58). MSCs demonstrate immunomodulatory properties through the inhibition of inflammatory reactions and the facilitation of anti-inflammatory pathways (59, 60). MSCs have the potential to enhance the production of anti-inflammatory cytokines, including interleukin-4 (IL-4), interleukin-10 (IL-10), and tumor necrosis factor β (TNF-β). Conversely, MSCs have the potential to decrease the production of inflammatory cytokines such as interleukin-1 (IL-1), interferon γ (IFN-γ), tumor necrosis factor α (TNF-α), and membrane cofactor protein-1 (MCP-1) (61–64). MSCs modulated various pathways related to immune cells and immune responses by controlling the levels of cytokines, thereby mitigating inflammation (65–67). Studies showed that TNF-α and IFN-γ were the central pro-inflammatory cytokines. Prostaglandin E2 (PGE2) has been identified as a significant mediator of MSCs, playing a crucial role in modulating the immune response and inflammation. It achieves this by regulating immunity, suppressing T-cell proliferation, and influencing T-cell differentiation (68). Following a stroke, there was a noted decrease in the level of PGE2, which subsequently increased after the transplantation of MSCs. This was accompanied by a reduction in the secretion of TNF-α in dendritic cells (DCs) and a reduction in the secretion of IFN-γ in T helper one cells and natural killer (NK) cells. Consequently, there was a notable decrease in the density of TNF-α, suggesting that MSCs mitigated the neuroinflammation induced by stroke (69, 70).

Studies conducted in laboratory settings have shown that MSCs play a role in stimulating the growth of neurogenesis and angiogenesis. MSCs release growth factors like vascular endothelial growth factor (VEGF) and brain-derived neurotrophic factor (BDNF), which contribute to the development of neurons and the creation of new vascular structures (71–73). These properties enable MSCs to mitigate tissue damage and enhance functional recovery in conditions such as TBI (6, 74).

MSCs are a kind of immunodeficient cell that is easily obtained. Allogeneic gene transplantation often does not result in immunological rejection. Human leukocyte antigen (HLA) class I is represented at modest levels in the majority of stem cells, according to earlier research. HLA class II is not expressed by them, nor are co-stimulator factors (CD40, CD80, and CD86) or surface markers of hematopoietic cells (CD34, CD45, CD79, and CD14) expressed by them (75–77). This characteristic allows stem cells to have immune privilege without provoking the host and transplanted cells' immune systems to clash (78). HLA class I is essential because modest protein concentrations can shield cells from the cytotoxic effects of NK cells (79). It has been documented that exposure to the pro-inflammatory milieu of injured tissues causes MSCs to express HLA class II (78). It has been shown that MSCs are extremely immunogenic after they are transplanted into the host (80). Over 90% of undifferentiated MSCs demonstrate the expression of HLA class II upon exposure to IFN-γ (81). Furthermore, to evade immune monitoring, hair follicle stem cells downregulate major histocompatibility complex (MHC) class I in the static state, according to research by Agudo et al. (82). Numerous variables, such as the microenvironment and cell state, might affect how immunogenic MSCs become. Consequently, more research on the specifics of MSC immunogenicity is required to increase the effectiveness of MSC transplantation (83).

MSCs exhibit various cell surface immune markers, leading the International Society for Cellular Therapy (ISCT) to establish identification criteria for MSCs in 2006. These criteria include characteristics such as plasticity and adherence, as well as the presence of CD73, CD90, and CD105 markers while lacking CD14, CD34, CD45, CD11b, CD79α, CD19, and HLA-DR markers. Additionally, MSCs should demonstrate the ability to differentiate into chondrocytes, osteoblasts, and adipocytes (84). The goal of the ISCT recommendations is to encourage cooperation among researchers and standardize MSC research. The common immunophenotypes of MSCs may generally be expressed by MSCs derived from various tissue origins, while the expression of the other immunophenotypes varies somewhat. As research develops and new information becomes available, this standard will likely be changed in the future (83).

### 2.1 Types of MSCs

Various origins of MSCs have been identified in the literature. Contemporary studies indicate stem cells can be isolated from diverse tissue sources (Figure 1). Bone marrow-derived mesenchymal stem cells (BM-MSCs), Human umbilical cord mesenchymal stem cells (HUC-MSCs), Adipose tissue-derived mesenchymal stem cells (ADSCs), and placenta-derived mesenchymal stem cells (PD-MSCs) are among the more researched MSC types. BM-MSCs are a diverse population of cells comprising pluripotent adult stem cells that can differentiate into many lineages, such as chondrocytic, adipocytic, or osteocytic (85, 86). This cell type constitutes ~0.001%−0.01% of bone marrow mononuclear cells (BMMNCs). It is characterized by the presence of CD73, CD90, and CD105 markers while lacking the expression of CD14, CD45, CD34, CD11b, CD79α, CD19, and HLA-DR surface molecules (87). Owing to its low abundance, large-scale *in vitro* culture and amplification are needed to provide enough samples for study or therapeutic application (88). BM-MSC collection is frequently an expensive and intrusive procedure. Additionally, when donor age increased, the quality of the BM-MSCs' cells declined noticeably (89, 90).

HUC-MSCs were isolated from Wharton's Jelly, a gelatinous connective tissue surrounding the umbilical cord blood vessels (91). Since it is often abandoned at birthing, collecting it is non-intrusive and presents few moral dilemmas (92). Its traits include a lengthy survival duration, a low doubling time, and potent anti-inflammatory properties. Prolonged *in vitro* culture has minimal effect on its genetic stability and phenotype (93–96). In contrast to BM-MSCs, HUC-MSCs exhibit enhanced proliferative capacity and reduced HLA-ABC and HLA-DR expression (97).

ADSCs are abundant in tissue reservoirs and can be acquired through minimally invasive procedures involving extracting subcutaneous white adipose tissue from the abdominal region, thighs, or buttocks of both animals and humans (98). ADSCs are easy to isolate and have a high yield-1,000 mL of adipose tissue can generate about 100 mL of ADSCs (99). It can differentiate into several lineages, such as hepatic, neurogenic, chondrogenesis, osteogenesis, cardiomyocyte, and adipogenesis (100, 101). In low-passage cultures, ADSCs frequently express CD34; however, this diminishes with ongoing cell passage (102, 103). In contrast to BM-MSCs, ASCs lack the expression of the sialoglycoprotein podocalyxin (PODXL) and the adhesion marker CD106 (104, 105).

The amniotic membrane, amniotic fluid, chorionic villi, chorionic plate, decidua basalis, entire placenta, and complete placenta are among the tissue sources of placenta-derived MSCs (PD-MSCs) (106). Compared to other tissue-derived MSCs, the placenta's stem cell-like cells have a greater capacity for differentiation and self-renewal (107). Furthermore, research conducted *in vivo* and *in vitro* has demonstrated its low immune qualities (108). Additionally, it has been shown that PD-MSCs promote monocyte differentiation from inflammatory M1 macrophages to M2-like macrophages, indicating that PD-MSCs may help treat inflammatory disorders (109). Nonetheless, MSCS that are separated from various placental regions have modest variations in characteristics. The placental tissue, for instance, comprises two distinct individual tissues: the fetal and the maternal placental tissues. Compared to MSCs produced from maternal placental tissues, those derived from fetal placental tissues exhibit substantially greater proliferation ability (110).

Gingival MSCs (GMSCs) are derived from various sources, such as periodontal tissue, dental pulp, and gingival ligaments. GMSCs exhibit MSC-related cell surface markers such as CD73, CD90, CD105, and stromal cell antigen 1 (STRO-1), just as MSCs from other sources (111). Research has also revealed that GMSCs may transdifferentiate into ectoderm and endoderm cell lineages, including keratinocytes, endothelial cells, and nerve cells, in addition to having the capacity to develop into the three mesoderm lines of adipocytes, osteocytes, and chondrocytes (112, 113). Furthermore, GMSCs can modulate the immune system, have an anti-inflammatory effect, and stimulate macrophage development (114–116). Moreover, GMSCS have constant morphological and functional properties under increased passage, are homogeneous, multiply quickly, and are not carcinogenic (114).

From normal human labial minor salivary glands, scientists have isolated heterogeneous cell populations with mesenchymal and epithelial characteristics (117). The existence of human labial gland-derived MSCs (LGMSCs) in the oral mucosa's lamina propria was later verified (118). Wang and colleagues effectively recovered MSCs from mature female salivary gland cysts and used flow cytometry to detect the distinctive MSC expression markers, such as CD29, CD44, CD73, CD90, and CD105. Notably, the salivary gland epithelial markers (CD49f) and CD34, CD45, CD106, and CD117 were negative (119). Compared to other MSCs, LGMSCs show a better capacity for differentiating into salivary gland epithelioid-like cells. They also possess the capability for osteogenic and lipogenic differentiation. Its capacity for adipogenic differentiation is, however, inferior to that of ADSCs (120, 121). LGMSCs can also modulate immunological function, have a shallow glandular site, and are simple to acquire and grow *in vitro* (122–124).

Furthermore, MSCs obtained from organs including the liver and pancreas are being studied, opening up possibilities for MSC multi-source routes. Noteworthy, MSCs derived from donors with type 1 diabetes mellitus (T1DM) have phenotypic and functional similarities with those of donors in good health. They can continue to perform secretory or immunomodulatory tasks typically (125). Nonetheless, MSCs derived from donors with type 2 diabetes mellitus (T2DM) frequently exhibit elevated apoptosis and senescence to reduced angiogenesis capacity (126).

### 2.2 MSCs and their extracellular vesicles (EVs)

The therapeutic benefits of MSCs may be attributed to the production and dissemination of EVs, although other substances released by MSCs are also linked to their therapeutic properties (Figure 1) (127–130). Owing to the lack of agreement over specific EV subtype markers, it is advised to use physical EV features like size (131–133). Consequently, EVs with <200 nm are called tiny EVs. Considering that microvesicles range in diameter from 100 to 1,000 nm, some of them may also be tiny EVs (131). Therefore, it won't be entirely proper to use the word “exosome.” The International Society for Extracellular Vesicles supports the term “EVs” to refer to any naturally occurring particles that are discharged from cells and are surrounded by a lipid bilayer but are unable to multiply due to the lack of a functioning nucleus (132, 133). Nearly all cells can release EVs, which are composed of many functional components, including proteins, lipids, enzymes, cytokines, receptors on the cell surface, and nucleic acids such as DNA, messenger RNAs (mRNAs), and microRNAs (miRNAs) (133, 134). EVs are essential for intercellular communication because, in their capacity as regenerative medicine, they deliver their payload to recipient cells (135, 136).

The issues with MSC use, particularly in human treatments, have made it possible to do related research on vesicles. Many of the therapeutic actions of MSCs were recapitulated by MSC-EVs, with notable enhancements. Recent research has demonstrated that MSC-EV therapies offer comparable or even greater efficacy than MSCs in treating a wide range of illnesses, all while lowering hazards significantly. Therefore, EVs took the place of their parent cells for several therapies. These demonstrations have created opportunities for a novel therapeutic approach based on the usage of MSC-EVs, which is commonly referred to as cell-free treatment (137). There are several benefits to this advancement, including quicker tissue penetration and more excellent safety. Additionally, the limited potential of MSC-EVs to trigger the immune system prevents disappointments even during allo- and xeno-grafts; the ease of transport and storage makes the potential of EV therapy optimal when compared to standard cell-based approaches; and the inability of MSC-EVs to self-replicate dramatically reduces the risk of tumors and expansions, typical of MSCs (137–141). Some clinical trials have developed as a result of these findings. Since MSC-derived EVs eliminate many of the hazards associated with MSC-based treatment, their applications are gaining popularity (142). The heterogeneity of MSC-EVs is an important characteristic. Their parent cells are produced in the stroma of tissues, which might vary, as was previously indicated (142).

## 3 Therapeutic effect of MSC on different brain injuries

### 3.1 Ischemic stroke

Stroke is the leading cause of permanent disability and the second leading cause of death worldwide, with ~5.5 million deaths per year (143). About 80% of stroke events include ischemic stroke (144). While TBI is often caused by motor vehicle or sports accidents and is the leading cause of death and disability in adolescents and young men, stroke mainly affects the elderly (145). Neonatal/perinatal ischemic stroke is a devastating disease that occurs once in every 3,500 births per year in the United States (146, 147). The consequences of perinatal stroke include spasticity, cognitive impairment, and death (148). Angiopathy and thromboembolism caused by intracranial or extracranial vessels are among the causes of ischemic stroke, and the most common cause in children under 15 years old is cerebral arteriopathy, which includes half of the cases (146, 148, 149). In stroke, rapid activation of innate immunity is the cause of inflammation (150). Breakdown of transcellular ion gradients due to the reduction of oxygen and energy supply, cytotoxic edema, production of toxic free radicals, and progressive thrombus formation in cerebral microvessels due to endothelial dysfunction are among the first pathological events after cerebral stroke (151). Studies have shown that CD4+ CD28-T cells increase in clinical conditions of acute ischemic stroke. T cells producing high amounts of γ-interferon and TNF-α probably have a direct pathogenic role in nerve damage (152). There are few treatments for ischemic stroke. In adults, the only FDA-approved drug for the treatment of ischemic stroke is tissue plasminogen activator (tPA), a thrombolytic agent. In comparison, the efficacy and safety of tPA in children are unknown (148). The paraspinal administration of etanercept in a study that included 629 patients with chronic neurological, mental, and clinical disorders after a stroke caused a partial improvement of the disease (153). The hypothesis that inhibiting the production of pro-inflammatory cytokines may be a therapeutic approach to treating brain injury is also proposed (154). Cytokines TNF-α, IL-1, and interleukin-6 modulate tissue damage in ischemic stroke (155). For the treatment of increased intracranial pressure (ICP) resulting from various causes, particularly ischemic and traumatic brain injuries, decompressive craniectomy has been employed. Given the rigid structure of the skull, brain swelling induced by stroke or TBI can lead to compartment syndrome and elevated ICP, necessitating this surgical intervention (156). Due to the little-known treatment methods, there is an urgent clinical demand for new treatment options. The therapeutic strategy can be successful in this situation if it targets several pathophysiological mechanisms that occur in different stages of brain damage (Figure 1) (143).

### 3.2 MSCs improve stroke outcomes by stimulating angiogenesis, neurogenesis, and synapse formation

MSC may play a role in the recovery of nerve damage by regulating various mechanisms such as immune system function and nutritional factor secretion (157–160). The mechanisms of action of MSCs have two levels: the peripheral level and the central level. The peripheral level includes the reduction of inflammation and immune modulation, and the central level is affected by angiogenesis, astrocytes, neurogenesis, axons, and oligodendrocytes (Figure 1) (18, 161). These cells will probably be able to create an environment that stimulates angiogenesis and neurogenesis, and on the other hand, they will increase the secretion of growth factors (162). Transplantation of MSCs into animal models of infants suffering from ischemic stroke improves performance. This mechanism works by stimulating neurogenesis, oligodendrogenesis, and axon regeneration. Infants are believed to benefit more from cell therapy than adults because infants have more flexible brains and different injury pathophysiologies. Also, microglial activation is more evident in infants because microglial activation is also present during the physiological development of the brain (153). The role of EVs derived from MSC is to prevent microglia activation (22).

An increasing body of preclinical research indicates that stem cell therapy shows promise in the treatment of ischemic brain injury and in mitigating its enduring consequences. The positive outcomes observed in phase I clinical trials of stem cell therapy for stroke have bolstered the confidence of researchers and clinicians in the potential clinical utility of this therapeutic approach (163, 164). Further improvement is necessary regarding the clinical applicability of these treatments and to validate their effectiveness and safety (165, 166). Additionally, the National Institutes of Health Consortium's “Stem Cell Therapies as an Emerging Paradigm for Stroke (STEPS)” has established the fundamental guidelines supporting the use of MSCs in clinical trials for stroke patients to guarantee the resolution of ethical, technical, and medical issues before clinical translation (167, 168). According to STEPS, human trials might involve either an immediate infusion of stem cells to reduce the chance of ischemia harm occurring later on or a late intervention to promote neuronal regeneration during the chronic phase of the stroke (164). Furthermore, additional data from earlier clinical studies has shown the necessity of enhancing critical elements, such as the proper selection of appropriate cells and the mode of delivery, to successfully convert preclinical findings into practical clinical practice (169).

After confirming that stem cell transplantation in stroke patients is a safe and well-tolerated treatment, higher-stage clinical trials sought to determine whether stem cells may offer quantifiable advantages (170). A randomized controlled trial (RCT) was conducted to examine alterations in neuroimaging metrics following the administration of stem cell-based therapy in individuals diagnosed with ischemic stroke. The participants were segregated into groups receiving MSC treatment and control groups. The neuroimaging assessment encompassed 31 patients who received MSC treatment and 13 control patients. Motor function was assessed through the Fugl-Meyer assessment scale. At the same time, neuroimaging techniques were employed to analyze fractional anisotropy in the corticospinal tract and posterior limb of the internal capsule, as well as connectivity within the motor network. The group receiving MSC treatment demonstrated a notable enhancement in motor function and preservation of corticospinal tract integrity, in contrast to the control group, which showed a deterioration in these aspects. Moreover, the MSC group exhibited heightened interhemispheric and ipsilesional connectivity, demonstrating notable variations in interhemispheric connectivity alterations compared to the control group. These results imply that stem cell-based treatment can promote network reconfiguration and prevent degeneration of the corticospinal tract, promoting motor recovery following a stroke (NCT01716481) (171).

A study conducted at a single medical facility, known as ISIS-HERMES, utilized an RCT design with an open-label approach to investigate the safety, feasibility, and effectiveness of intravenous (IV) autologous BM-MSCs in individuals aged 18–70 who had experienced moderate to severe ischemic carotid stroke within 2 weeks of its onset. The study had a follow-up period of 2 years. Participants were assigned randomly in a 2:1 ratio to receive MSCs or standard care. The main objectives of the study were to evaluate the feasibility and safety of the intervention, with an additional focus on secondary outcomes, such as overall improvement and motor recovery, assessed through fMRI during passive wrist movements. Out of 31 participants, 16 were administered MSCs, demonstrating a treatment feasibility rate of 80%. The cohort that received MSC treatment exhibited notable enhancements in motor-NIHSS (*p* = 0.004), motor-Fugl-Meyer scores (*p* = 0.028), and task-related fMRI activity within the primary motor cortex regions MI-4a and MI-4p (*p* = 0.031 and *p* = 0.002, respectively). The findings suggest that administering IV autologous MSC therapy following a stroke is both safe and viable, and it contributes to improved motor function recovery by promoting sensorimotor neuroplasticity (NCT00875654) (172).

A phase 2 single-center, assessor-blinded RCT investigated the safety and efficacy of IV autologous BM-MSCs in 17 patients aged 30–75 with severe ischemic stroke in the middle cerebral artery territory. Participants were randomly assigned to receive either BM-MSCs or conventional treatment. The primary endpoints evaluated after 12 months were the National Institutes of Health Stroke Scale (NIHSS), modified Rankin Scale (mRS), Barthel Index (BI), and MRI infarct volume. The findings indicated no notable variations in NIHSS, mRS, or BI among the groups; however, a marked decrease in median infarct volume was observed in the BM-MSC group. The treatment was deemed safe and well-received, indicating promising advantages in diminishing infarct volume (NCT01461720) (173).

Chung et al. (174) conducted an RCT to investigate the potential benefits of autologous-modified MSCs in enhancing recovery among individuals with chronic stroke. The study's findings indicated that the IV administration of preconditioned autologous MSCs, along with autologous serum, was both feasible and safe for patients with chronic severe stroke. Additionally, the researchers noted improvements in foot movement through detailed functional assessments (NCT01716481) (174). These clinical trials collectively emphasize the therapeutic potential of MSCs in improving stroke outcomes.

### 3.3 Brain hemorrhages

Spontaneous ICH is one of the most detrimental cerebrovascular diseases globally, causes immoderate morbidity and mortality, and is a kind of stroke. Cerebral hemorrhage is frequently categorized based totally on the same region in the brain where it happens (175). Bleeding within the brain itself is called ICH. Bleeding can also occur between the lining of the brain and the brain tissue itself. That's known as a subarachnoid hemorrhage. If a blood clot occurs between the cranium and the brain, it's known as a subdural or epidural hematoma, depending on whether it's far underneath or over the difficult overlaying (dura) of the brain. Subdural and epidural hematoma most likely occur due to stressful brain damage or after a fall (176). The pooled blood that creates a hematoma within the brain can cause extended intracranial stress, which in turn damages the mind's parenchyma and may result in everlasting nerve damage or loss of life. The most common cause of cerebral hemorrhage is high blood pressure. Over time, excessive blood strain can weaken the arterial walls and lead to rupture, which is set at 13% of strokes, hemorrhagic strokes, or spontaneous bleeding within the brain (176). The reasons for cerebral hemorrhage consist of high blood stress (high blood pressure) or cerebral amyloid angiopathy (CAA), which is one of the most common and important reasons for cerebral hemorrhage. With the passage of time and age, blood pressure will increase and may harm the walls of cerebral arteries. Weaken and expand (aneurysm) abnormally and lead to rupture (177–179).

MSCs have shown promising results in preclinical models for the treatment of ICH. MSC treatment has improved neural network reconstruction, neurological functioning, and ICH-induced neuronal abnormalities through anti-inflammatory, neurogenesis, angiogenesis, and anti-apoptotic effects (180, 181). By modulating immune responses and releasing anti-inflammatory cytokines, MSCs lessen the inflammatory cascade set off by brain hemorrhage. This reduces the possibility of further harm to brain tissue (182, 183). A study conducted by Azevedo et al. (184) in a preclinical setting aimed to examine the possible effects of MSCs on CD4 T cells. The findings indicated that MSCs prompted the differentiation of CD4 T cells into regulatory T cell (Treg)-like cells through the activation of TGF-β and, or programmed death-1 (PD-1)/and programmed death ligand 1 (PD-L1) signaling pathways. Experimental evidence has confirmed that PD-L1 downregulates the migration of CD4+ T cells to the brain, leading to the upregulation of Th2 and Treg cells while simultaneously downregulating Th1 and Th17 cells. This regulatory effect is mediated through the mTOR pathway, as demonstrated in both *in vitro* and *in vivo* studies (185). Additionally, other research has shown comparable findings in the ICH rodent model, a specific B10.D2 [H-2(d)) donor to BALB/c (H-2(d)] recipient mice model, and the experimental autoimmune neuritis (EAN) rat model (185–187). It is widely recognized that neuroinflammation exacerbates the advancement of brain damage resulting from ICH. Therefore, interventions to modulate the immune response can potentially mitigate ICH-induced brain injury. The notable characteristics of anti-inflammatory and immunomodulatory effects render MSC transplantation a suitable therapeutic option for addressing inflammatory conditions such as ICH. This is achieved by modulating microglia and neutrophils, augmenting the defensive role of anti-inflammatory cytokines, and mitigating the adverse effects of pro-inflammatory cytokines (181, 188, 189). Kim et al. (190) discovered that transplanting ADMSCs into rats with an ICH model resulted in a reduction of acute inflammation and chronic brain deterioration, leading to enhanced long-term functional recovery (190).

BMSCs are commonly employed in treating brain injuries due to their convenient procurement from the host and ability to penetrate the blood-brain barrier (BBB) without causing structural disruption. This allows them to differentiate into neurons or neuron-like cells, facilitating tissue repair (191–195). Several research studies have shown that BMSCs have the potential to reduce neurological impairments and maintain the integrity of the BBB in rats with ICH (182, 196). In their study, Chen et al. (197) observed that using rat ADSCs in treating rats with ICH resulted in the development of cells resembling neurons and astrocytes near the injury site. Additionally, this treatment led to enhanced levels of VEGF, contributing to the restoration of neurological function in the affected rats (197). Yang et al. (198) utilized ADSCs obtained from the fat tissue of a 65-year-old male donor and administered them via injection into the right femoral vein of rats with ICH-induced stroke. Their findings indicated that transplantation of ADSCs may enhance the functional recovery of the test subjects (198).

MSCs reduce brain damage after ICH through the Hippo signaling pathway, which could promote neurogenesis and decrease the facet outcomes of intellectual injuries (199, 200). The Hippo signaling pathway is regulated by kinase activity, specifically involving mammalian sterile 20-like kinase 1 (MST1) and its associated protein, Yes-associated protein (YAP), which protects astrocytes from apoptosis. This pathway induces nuclear translocation of YAP by suppressing MST1 using small interfering RNA (siRNA). Furthermore, studies suggest that astrocytes adopt an astroglial-mesenchymal phenotype after BM-MSC transplantation, potentially enhancing their reparative capacity through the Hippo pathway. These findings position the Hippo signaling pathway as a promising therapeutic target for advancing the treatment and management of ICH (199, 201, 202).

### 3.4 Concussions

A concussion is a “temporary disturbance in brain function as a result of trauma” and is a subset of neurological accidents referred to as annoying brain accidents. Concussions are technically a subset of mild traumatic brain injury (mTBI) (203). Concussions arise as a result of direct or oblique trauma to the head. However, indirect effects from forces someplace else inside the body can bring about acute acceleration or deceleration harm to the brain, which can also result in concussions (204). A complex web of biological processes, including structural modifications, neurochemical shifts, and functional deficits, interact to cause concussions. Cellular and metabolic alterations may result from the first impact's ability to stretch and damage axons. These consist of inflammatory reactions, disruption of the BBB, and excitatory neurotransmitter release. All of these mechanisms have a role in the acute and long-term symptoms that people with concussions experience. The discharge of electrolytes through ion channel depolarization results in the release of neurotransmitters and subsequent neuronal dysfunction. Adjustments in glucose metabolism lower cerebral blood flow, and mitochondrial dysfunction additionally occurs (205, 206).

MSCs and their EVs have emerged as promising therapeutic agents for addressing the complex pathophysiology of concussions (207, 208). A 2013 experiment that included 97 TBI patients who received autologous BM-MSCs by lumbar puncture lends support to the safety and effectiveness of this cell treatment in the context of clinical investigations of MSC therapy for TBI. After receiving a transplant, over 40% of patients showed better neurological function. Twenty-seven of the 73 individuals initially presented with motor problems showed improved motor abilities. The study found that patient age and the administrative window following injury all impacted the result, with younger patients responding better to the advantages of a cell transplant (209). Because of the features of multi-capacity and self-renewal and their availability and occasional immunogenicity and ability after freezing, their use turns them into a promising treatment for injuries and strokes. MSCs are multipotent stem cells with the potential for self-renewal and more than one differentiation (210–214). Inside the mouse TBI model, IV-injected BM-MSCs can penetrate the BBB and introduce dietary elements into the mind enhancer. They can also selectively switch them to the damaged areas of the brain tissue and differentiate them into neurons and astrocytes (215, 216). Advertising of axonal regeneration within the mind and angiogenesis and increasing glial cells on the site of harm can boost the internal restoration procedure. Additionally, MSC-derived EVs, which carry bioactive molecules such as mRNA, miRNA, and anti-inflammatory cytokines, play a crucial role in intercellular communication and neuroprotection (217). EVs derived from BM-MSCs prompt T cells by freeing anti-inflammatory cytokines and affecting apoptosis (218, 219). Research displays that EVs decreased using MSCs in hypoxic situations can put off neuronal degeneration and cause neurological recovery (220). Therefore, research is needed to determine the quality of MSCs to treat TBI. Thinking about the prevalence of mitochondrial dysfunction in TBI, enhancing mitochondrial features has been a practical therapeutic aim for acute brain harm in recent years. Mitochondrial switching from MSCs can lessen the rate of apoptosis in recipient cells and enhance cellular survival by regulating the Bcl-2-associated protein X (Bax)/Bcl-2 ratio (221). MSCs can also boost the expression of the antiapoptotic gene Bcl-2 and decrease the extent of superoxide anion, thereby shielding brain tissue (222). This mitochondrial transfer occurs among MSCs and target cells via tunneling nanotubes (TNTs), microvesicles, EVs, hole junctions, and cytoplasmic fusion (223–226). Many studies have shown that MSCs can shield brain tissue from excessive harm by inhibiting oxidative pressure. In a TBI mouse model, overexpression of specific genes, including that for superoxide dismutase 2, *in vitro* can enhance the antioxidant impact of MSCs and improve their therapeutic effects (227). In conclusion, while concussions pose significant clinical challenges, MSCs and their EVs offer innovative therapeutic strategies. Much medical research is underway to decide the most advantageous course and timing of management and dosage of MSCs and EVs, which can be famous directions for future studies.

### 3.5 Tumor

Tumors that form in the brain can have debilitating effects, even if the tumor is benign (228). Glioblastoma multiforme (GBM) is the most common and aggressive brain tumor and a complex and resistant cancer. GBM can originate from normal brain cells or low-grade astrocytes (229, 230). Although the use of surgery, radiotherapy, and chemotherapy is suggested to increase life expectancy and quality of life, there is currently no cure for this cancer (231). One of the ways that has created new hope for the treatment and reducing the complications of GBM is mesenchymal stem cell therapy (232).

Studies have shown that MSCs are toxic to tumor cells. Also, new research shows that these cells induce apoptosis through the phosphatidyl-3-kinase/protein kinase B (AKT/PI3K) pathway and suppress the growth and proliferation of glioma cells (233). MSCs have anti-angiogenic properties, which has made them sound like anti-tumors; observations show that these cells can inhibit tumor blood vessels (234). Without forming a new ship and access to blood, tumors cannot grow more than 2–3 cubic millimeters (235). The process that controls and inhibits angiogenesis by MSCs in GBM is the reduction of focal adhesion kinase (FAK) and integrin β2α expression. FAK is a cytoplasmic tyrosine kinase involved in integrin activation, regulation of cell migration, proliferation, persistence, and aggression play a role. Research shows that MSC reduces FAK activity and reduces the formation of new blood vessels in the tumor (236). MSCs can induce apoptosis by inducing glioma cell death by downregulating X protein-associated inhibitor of apoptosis (XIPA). This protein is a member of the family of apoptosis inhibitor proteins, which has a positive effect on most malignancies, including GBM (237). MSC prevents the increase of glioma cell lines, accompanied by a 50% increase in cytotoxicity and apoptosis. The data obtained from the western blot shows a significant reduction of XIPA and alpha-serine/threonine kinase, which is associated with cell death. Further studies show that huc-MSC MSCs express genes encoding pro-proteins. Belonging to the pro-apoptotic Bcl-2 family, including Bax Bad, these studies confirmed the successful induction of apoptosis by MSC, huc-MSC, in glioma cells (238). An additional potential anti-tumor mechanism associated with MSCs is their influence on epidermal growth factor receptor (EGFR) signaling (239).

A few of these tactics have been taken to the clinic, where at least two ongoing clinical trials assess the potential benefits of MSC-based GBM therapies (240). The initial study, conducted at the M.D. Anderson Cancer Center in Texas, United States, is an extension of their findings from preclinical experiments involving GBM. In these experiments, allogeneic MSCs carrying oncolytic viruses (OVs) were introduced into the carotid artery, demonstrating encouraging outcomes (NCT03896568) (241). In this phase I clinical trial, an open-label approach is employed to investigate the utilization of the conditionally replicating oncolytic adenovirus Delta24-RGD in conjunction with MSCs. This strategy aims to capitalize on the inherent tumor-tropism of MSCs, with the potential benefit of restricting the dissemination of the virus to non-target organs. Additionally, this enables MSCs to penetrate the BBB and disperse broadly throughout the tumor (NCT03896568). Patients with recurrent glioblastoma are being treated in another research at CHA University in South Korea by transplanting MSCs that express the suicide gene cytosine deaminase (CD). This phase I/II clinical trial is open-label and aims to assess safety, efficacy, and maximum tolerated dosage (NCT04657315).

### 3.6 Immune modulator by MSCs

There is a similarity between macrophages and MSCs, such as the ability to switch between pro- and anti-inflammatory phenotypes. Stefani and colleagues tested the effect of low-dose MSCs on GBM. The GL261 glioma cell line implanted in the right striatum of mice treated with irradiated BM-MSCs increased mouse lifespan and reduced tumor volume by ~67%, and histochemical staining of the vessels of the tumor mass also showed that the blood vessels inside the tumor had decreased density (238). One of the most essential characteristics of malignant gliomas is the immunosuppression that an active tumor creates in the existing tissue (242). Therefore, one of the anti-cancer treatment strategies based on MSCs is dependent on intra-tumor immunomodulating cytokines, such as ILs. Momeh et al. researched that intra-tumor administration of genetically modified MSCs increased expression. IL-7 and IL-2 control pro-inflammatory intracranial tumors. Recent studies have proven that MSCs that express IL-24 secret immunomodulatory cytokines suppress tumor growth and induce apoptosis of glioma cells (139). Other cytokines delivered by MSC-dependent strategies include interferon-beta (IFN-β) secretion (243). MSCs represent a groundbreaking approach to GBM treatment by targeting tumor growth, angiogenesis, and the immunosuppressive microenvironment.

### 3.7 Encephalitis

Encephalitis means brain inflammation. This disease is mainly caused by viruses and, in some cases, due to the immune system. The most common viral encephalitis is caused by herpes simplex infection, and other causes are viruses such as rabies, polio, and measles (244–246). Adults with encephalitis manifest with the onset of high fever, headache, confusion, and sometimes seizures. Younger children or infants may present with irritability, loss of appetite, and fever (247).

Currently, the application of MSCs in treating encephalitis demonstrates significant potential (248–250). MSCs have characteristics such as the ability to regulate immunity, the ability to migrate to the site of injury, and the ability to differentiate into different types of cells such as fat cells, osteocytes, chondrocytes, and neuron-like cells (95, 251, 252). Research shows that MSC transplantation can regulate the expression of BDNF and nerve growth factor (NGF) and also can improve nerves in many central nervous system (CNS) diseases (253–256). By regulating inflammation and other processes, MSCs have a therapeutic effect on most CNS diseases. In recent years, many studies have been conducted on the therapeutic effect of MSCs on CNS and viral diseases (257–261). In the research, they found that MSC transplantation improved the life span and also reduced the neurological symptoms in the mouse model that was infected with encephalitis and also in the mice that are suffering from neuroinflammation and treated with MSC, based on the changes in Pathological tissue, their neuroinflammation decreases. Research in the laboratory shows that IV or intraspinal administration of MSC improves the autoimmune encephalitis (EAE) mouse model and causes the disease to decrease significantly. Mice infected with JEV without MSC treatment showed clinical signs of encephalitis, which starts with piloerection and physical limitations, followed by paralysis and stiffness, and finally leads to severe neurological symptoms such as paralysis, seizures, and even death. The group treated with MSC had a faster recovery in terms of weight and behavioral conditions. Also, the lifespan in this group increased significantly compared to the group that was not treated, and studies have shown that treatment with MSC also reduces pain. Severe meningitis decreased significantly in JEV-infected mice treated with MSCs, and the levels of inflammatory cytokines and chemokines were also reduced in this group compared to the untreated group (261). Experiments show that after JEV infection, either *in vivo* or *in vitro*, a significant amount of TNF-α is produced, and MSCs can produce TSG-6 (TNF-α-stimulated gene/protein 6); by inducing TNF-α, it moderates inflammatory responses, controls BBB destruction, and also improves tissue damage. Through the experiments they conducted, researchers found that the expression of cytokines transforming growth factor TGF-β and TSG-6 in MSCs that were Cultured with Neuro2a cells that were infected with JEV was increased, which has an anti-inflammatory role (262, 263).

MSCs, in addition to their role in regulating inflammation, possess a protective immune function against various injuries caused by bacteria and viruses (259, 260, 264–268). Research indicates that MSCs exhibit an antiviral role. Also, the titer of JEV decreased in Neuro2a cells cultured simultaneously with MSC. One of the reasons for this is that MSCs can improve innate and adaptive immune responses by modulating immunity and helping eliminate the virus (259, 269, 270). Neuro 2A (N2a) is a cell line derived from mouse neural crest cells that is widely utilized in research focused on neuronal differentiation, axonal growth, and various signaling pathways. A notable feature of these cells is their capacity to undergo differentiation into neurons within a matter of days (261).

Critical to the efficacy of MSC therapy for neurological disorders are the dosage, duration, and route of administration of MSCs. Various studies have utilized dosages ranging from 1 × 10^6^ to 5 × 10^6^ for each kilogram of body weight across different animal models and types of injuries. Furthermore, the optimal dosage remains to be determined, as variations in dosage may lead to differing therapeutic outcomes. Consequently, further investigation is essential to refine dosing strategies and maximize the therapeutic potential of MSCs (162, 253, 280).

The route of administration is another critical factor that affects the bio-distribution, retention, and therapeutic efficiency of MSCs. Different routes, including IV, IA, intrathecal, intraperitoneal, and localized injections, have been employed in numerous studies (271, 281–283). IV injection is frequently used in instances of extensive injury, including conditions such as stroke, TBI, and Parkinson's disease, to facilitate the widespread dissemination of cells throughout the body, encompassing the affected brain tissue (280, 284, 285). IA administration represents a promising route for delivering MSCs in the treatment of neurological disorders, particularly ischemic stroke and brain injuries (286, 287). Compared to intracerebroventricular, intraparenchymal, and IV stem cell administration, IA stem cell distribution after ischemic stroke is less invasive and permits better diffusion and distribution of more significant stem cells inside and outside the infarct area (288). Moreover, it mitigates the risk of MSC entrapment in the lungs and liver, a standard limitation of IV administration (289). For instance, Zhang et al. (290) demonstrated that IA delivery of bone marrow MSCs resulted in the most significant neurological recovery compared to IV and intracerebral routes in a rat model of cerebral ischemia (290). Thus, a thorough understanding of MSC dosage and administration routes is critical for optimizing therapeutic outcomes and advancing MSC therapy from preclinical studies to clinical applications.

## 4 MSC-derived extracellular vesicles (EVs) as therapy for TBI

### 4.1 Neurorestorative effects of MSC-EVs

**Figure 2:**
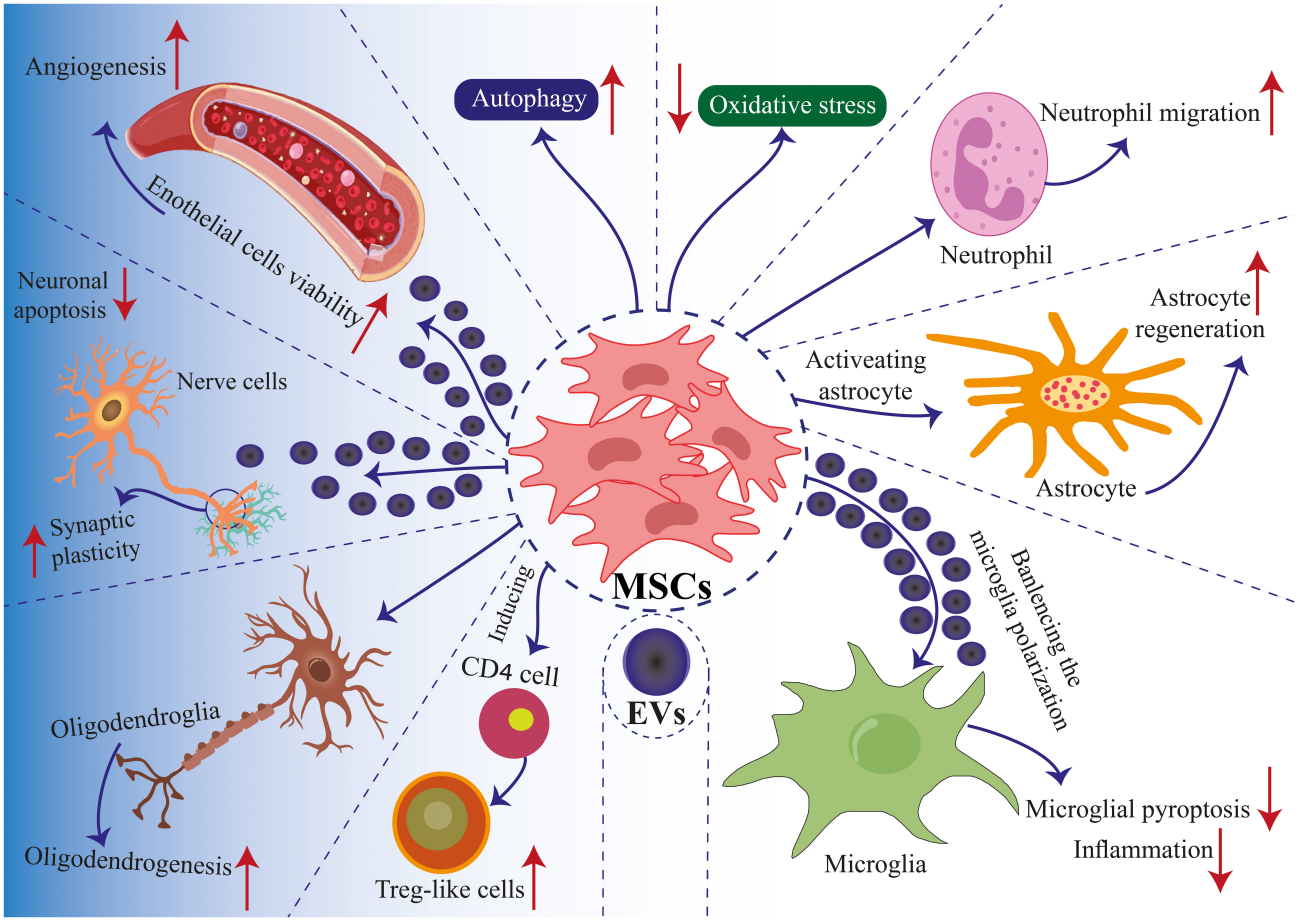
Mechanisms of MSC application in TBI. Communication between MSCs and tissue environments is facilitated through two primary mechanisms: direct cell-to-cell interactions and communication via EVs. MSCs engage in communicate with neighboring cells such as immune cells, nerve cells, glial cells, and endothelial cells, enabling regenerative healing and the architectural reorganization of injured tissue. MSCs produce EVs that encapsulate proteins, lipids, microRNAs, and cytokines, facilitating the transfer of functional molecules among cells. Positive modulation enhances biological functions such as autophagy, apoptosis, pyroptosis, inflammation, angiogenesis, cell plasticity, cell migration, and oxidative stress. These interactions induce the differentiation of MSCs into specific cell lineages and regulate immune cell reactions (181).

MSC-derived EVs have been shown to have neurorestorative capacity and have emerged as an innovative TBI therapy (Figures 1, 2). MSC-derived EVs have been demonstrated to enhance functional recovery in a rat model of TBI with postponed IV injection in a broad range of efficacious dosages (50–200 μg protein/rat) for TBI therapy with a prolonged therapeutic window from 1 day to 7 days post injury (291). In addition, EVs generated from monkey BM-MSCs that are given 24 h after an injury can improve fine motor function recovery in a monkey cortical injury model (292). The protective benefits of MSC-derived EVs in rats with TBI are mediated via endogenous angiogenesis and neurogenesis, as well as inflammation reduction (291). Following TBI, endogenous neurovascular plasticity, such as neurogenesis, angiogenesis, axonal sprouting, and synaptogenesis, occurs. This may aid in the brain damage's natural healing process (293). Post-brain damage, spontaneous healing is not always possible. They are developing innovative treatments to increase neurovascular plasticity and promote functional recovery following TBI, which is urgently needed. In the dentate gyrus of the damaged hippocampal brain, there is an increased endogenous neurogenic response in the subventricular and subgranular zones. This response is linked to the recovery of cognitive function following TBI (294). Neural stem cells located in the subventricular zone and subgranular zone exhibit the capacity to continually produce new neurons in adult mammals, which subsequently differentiate into fully developed neurons. The ability of adult-born dentate gyrus granule cells to integrate functionally into the current circuitry is well known (295). Neurogenesis and angiogenesis are markedly increased in the wounded brain following TBI when treated with MSC-derived EVs (beginning 24 h after injury), which may partially account for functional recovery following TBI (296). Normal brain vasculature is quiescent, but following an injury, it becomes active. Growth factors that support neurorestorative processes like neurogenesis and synaptogenesis may be secreted by activated vasculature, which might promote functional recovery following brain damage (297).

A thorough analysis has been done on the possible impacts of EVs produced from MSCs on neuroinflammation, neurogenesis, and, particularly, functional recovery in TBI (298, 299). According to a recent study, in elderly rats with a stroke, BM-MSCs-EVs boost post-stroke neurogenesis next to the subventricular zone and support functional neurological recovery (300). Through endogenous angiogenesis and neurogenesis promotion, MSC-derived EVs from human BM-MSCs (100 μg protein, IV) markedly enhance functional recovery following ICH in rats (301). Therefore, MSC-derived EVs without cells might be a potential treatment for ICH. The introduction of cell-free EVs derived from MSCs via IV administration following a stroke has been shown to enhance functional recovery and promote neurite restructuring, neurogenesis, and angiogenesis. This approach presents a novel therapeutic strategy for treating stroke (302–305). By defending the BBB, preventing apoptosis, reducing inflammation, and controlling autophagy in brain lesions through various chemicals and mechanisms, including miRNA, EVs may enhance cognitive function (306). *In vitro* neural progenitor cell neural development is promoted by EVs produced from adipose-derived MSCs (307). Through the transfer of miR-25 from EVs generated from adipose-derived MSCs, autophagy is decreased in stroke mice (308). In a stroke-prone mouse model, EVs derived from human BM-MSCs enhance neurodegeneration and avert post-ischemic immunosuppression (309). EVs from human BM-MSCs reduce neuroinflammation in rats with focal brain damage (310). Mice exposed to ischemia brain injury are protected against MSC-derived EV-enclosed microRNA-93 (311). EVs derived from HUC-MSCs, when administered intranasal, demonstrate neuroprotective properties and enhance functional recovery following perinatal brain injury in rat models (312). The above research indicates that EV therapy enhances functional recovery by affecting the immune system, neurogenesis, neurorestoration, angiogenesis, and neuroprotection.

Neuroinflammation is a distinguishing feature of both acute and chronic TBI. An emerging mechanism facilitating cell-cell communication in regulating immune responses involves EVs (313). After being injected IV, green fluorescent protein-tagged (GFP^+^) MSC-EVs can be absorbed by microglia, astrocytes, and neurons in the TBI rat brain as early as 30 min later. More EVs are found in the injured hemisphere, and GFP^+^ EVs are co-localized with CD68+ macrophages in the liver, spleen, and thymus (314). These findings imply that IV injection of EVs may play not only central functions in promoting neurovascular remodeling and controlling neuroinflammation but also non-central effects in modulating peripheral immune responses. If there are any non-CNS effects, they would be complimentary and would not lessen the therapeutic benefits of EVs on neurovascular remodeling and functional recovery (315, 316).

### 4.2 Neuroprotective effects of MSC-EVs

Giving MSC-EVs to mice as soon as possible (15 min after the injury) reduces the size of the lesion and enhances their functional ability. This is achieved by modifying the polarization of macroglia and microglia, boosting the expression of the anti-apoptotic protein Bcl-2, but decreasing the expression of the pro-apoptotic protein Bcl-2-associated X protein and pro-inflammatory cytokines, IL-1β and TNF-α (317). The neuroprotective effectiveness of MSC-derived EVs has been studied in large animal models in translational research. Early (1-h post-injury) single-dose treatment of MSC-derived EVs reduces brain swelling, lesion size, and BBB breach, providing neuroprotection in a combined swine model of TBI and hemorrhagic shock (318). These findings promote further research into EVs as a cutting-edge TBI treatment by showing that cell-free EVs have neuroprotective and neurorestorative benefits for enhancing TBI functional recovery. Utilizing MSCs from human BM as the source of EVs might guarantee the most significant translational potential for TBI research in big animals. Multiple citations regarding the utilization of human BM-MSCs-derived EVs in extensive animal models of TBI, specifically in monkeys and swine, are outlined in Supplementary (Table 2) (130, 292, 318–320).

**Table 2:** Treatment with EVs derived from MSCs in animal models of TBI.

**TBI model**	**TBI animal species/sex**	**MSC source for EVs**	**Dosage**	**Injection time/route**	**Conclusion**	**References**
	Mice/male	Human BM-MSCs	6.4 or 12.8 or 25.6 × 10^−9^ EVs/mouse	90 min PI/IN	Avoiding long-term mood and cognitive deficits in a dose-dependent manner	(325)
	Mouse/male	Rat bone mesenchymal stem cells (BM-MSCs)	30 μg protein/mouse	15 min PI/retro-orbital	Inhibiting neuroinflammation, reducing lesion size, improving neurobehavioral performance	(317)
	Mice/male		3.8, 7.5, 15, 30 μg EVs per mouse	1 h PI/IV	Restoring cognitive deficits following TBI and reducing neuroinflammation in a dose-dependent manner	(326)
CCI	Rat/male		50, 100, 200 μg protein/rat	One day PI/IV	Demonstrating a broad spectrum of safe dosages for the treatment of TBI with a minimum 7-day therapeutic window	(291)
	Rat/male	Human BM-MSCs	100 μg protein/rat	One day PI/IV	Enhancing native neurogenesis and angiogenesis, decreasing neuroinflammation, and improving functional recovery	(296)
	Rat/male		100 μg protein/rat	One day PI/IV	EVs produced from hMSCs cultivated in 3D scaffolds outperform EVs from hMSCs cultured in 2D conditions in terms of spatial learning results.	(327)
	Rat/male		100 μg protein/rat	One day PI/IV	Compared to naïve exosome therapy, miR-17-92 cluster-enriched EVs show superior therapeutic effects on improved functional recovery by lowering neuroinflammation and cell death and boosting angiogenesis and neurogenesis.	(328)
	Rat/male		100 μg protein/rat	One day PI/IV	MiRNA attenuation in EVs produced from MSCs eliminates the beneficial effects of EV therapy on TBI healing.	(329)
	Rat/not described	Rat BM-MSCs	100 μg protein/rat	1 h PI/IV	Minimizing neurological harm through the mitigation of glutamate-induced excitotoxicity	(330)
Feeney's weight-drop method	Rat/male	Human adipose MSCs	20 μg protein/rat	One day PI/ICV	As a result of their targeted entry into microglia and macrophages following brain damage, hADSC-EVs reduce inflammation and promote functional recovery.	(331)
CCI combined with hemorrhage shock	Swine/female	Human BM-MSCs	1 × 1012 particles/swine	1 hour PI/IV	Lowering blood-based cerebral biomarker levels, decreasing brain edema and lesion size, and enhancing BBB integrity	(318)
CCI combined with hemorrhage shock	Swine/female	Human BM-MSCs	1 × 1013 particles/swine	1 h PI/IV	Reducing inflammatory networks in the brain and encouraging neurogenesis and neuroplasticity	(332)
Cortical injury in the mapped hand representation.	Monkey/female	Monkey BM-MSCs	4 × 1011 particles/kg	One day and 14 days PI/IV	Enhancing recovery of motor function	(292)
Cortical injury in the mapped hand representation.	Monkey/male and female	Monkey BM-MSCs	4 × 1011 particles/kg	One day and 14 days PI/IV	Reducing hyperexcitability brought on by injuries and reestablishing excitatory/inhibitory balance in the ventral premotor cortex to return cortical networks responsible for motor function to normal	(320)

CCI, controlled cortical injury (an open head injury, focal contusion); hADSC, human adipose mesenchymal stem cells; IN, intranasal administration; IV, intravenous administration; PI, post-injury.

While we concentrated on the therapeutic benefits of MSC-derived EVs in TBI, EVs produced from a wide range of other cells, such as astrocytes, microglia, and neural stem cells, can also improve functional recovery in TBI (207). Adipose tissue is commonly regarded as refuse and disposed of; however, it represents a valuable reservoir of cells (321, 322). It has been demonstrated that adipose tissue is a rich, readily available, and plentiful source of adult stem cells with multipotent qualities appropriate for tissue engineering and regenerative medicine applications (321). Treating TBI using MSC-derived EVs has shown to be a promising approach (323, 324).

## 5 Potential challenges and limitations

Given their ease of separation, minimal immunogenicity, and capacity to develop into a variety of tissue lineages, including brain cells, MSCs provide the most therapeutic promise (281). Nonetheless, there are still several restrictions on MSC transplantation. MSCs are likely to be contaminated during their cultivation and treatment, and cells cultivated *in vitro* are susceptible to mutation. Foreign infections may potentially spread as a result of cell transplantation. Furthermore, MSC transplantation may provide cancer cells vitality and encourage the development and spread of tumors. It is unclear how MSCs initiate and control mitochondrial translocation. Furthermore, it is impossible to overlook the possibility of allogeneic immunological rejection. Therefore, enhancing MSC therapy's safety is very crucial (21).

In the following, we discuss some challenges and limitations of using MSCs in nervous system disorders and TBI.

### 5.1 Immunocompatibility

It's interesting to note that MSCs can have proinflammatory and immunosuppressive effects. These effects are contingent upon the degree to which the cell is stimulated by chemokines (e.g., PGE2, TGF-B, IL-6, IL-10, HLAG5), metalloproteinases, nitric oxide (NO), indoleamine-2,3-dioxygenase (IDO1), and inflammatory cytokines. Therefore, MSCs' immunosuppressive activity can be used to avoid instances of allograft rejection as well as an aberrant inflammatory or autoimmune response (333). MSCs are known to exhibit immunosuppressive effects in the presence of NO on a molecular level (334, 335). In contrast, in regions deficient in NO, specifically in areas where the activity of inducible nitric oxide synthase (iNOS) is suppressed, MSCs promote the proliferation of immune cells. Additionally, Qin et al. (336) have found that MSCs cannot stop T-cell proliferation in rat models when the NOS inhibitor L-NMMA is present (336). These results strongly suggest that NO generation or increased NOS2 activity is necessary for starting MSC-mediated immunosuppression. In MSC-mediated immunomodulation, indoleamine 2,3-dioxygenase is a switch similar to NO (337, 338). While some studies indicate that MSCs may play a role in cancer development, others show that they have a suppressive impact on the growth of tumors (339). The processes that underlie these suppressive effects include the induction of cell cycle arrest, suppression of proliferation-related signaling pathways PI3K/AKT, and, ultimately, a decrease in cancer development (340). On the contrary, alternative research has demonstrated that MSCs can differentiate into cancer-associated fibroblasts (CAFs) and consequently play a role in facilitating cancer advancement (341–343).

### 5.2 Stemness stability and differentiation of MSCs

Numerous factors, including the technique used for separation, the individual diversity of the source tissue, the donor's health, and the specific cell culture's history, might influence the stemness qualities of MSCs (344). Roughly 10% of the cells in the dental pulp are mesenchymal stem cells (DP-MSCs). Compared to BM-MSCs and AT-MSCs, DP-MSCs generate fewer proangiogenic factors *in vitro*, although having more excellent proliferation rates (345). However, additional research has demonstrated that the chemokines and neurotrophins that DP-MSCs release are essential for neuroprotection and the body's reaction to nervous system damage (346, 347). Remarkably, MSCs display donor-related differences as well. These may result from the patient's age, underlying illnesses, gender, body mass index (BMI), and donor place (344). In a rat model, MSCs extracted from female donors are more effective than MSCs from male donors in lowering lung inflammation, according to a 2016 research by Sammour et al. (348). On the other hand, the osteogenic capacity of local stem cells is diminished by the hormonal fluctuations that women experience, particularly after menopause (349). Additionally, Ogawa et al. (350) confirmed that there is gender variability in AT-MSCs by observing that cells produced from female mice had greater levels of the adipogenesis marker Peroxisome Proliferator-Activated Receptor-⋎2 (PPAR-⋎2) (348–350). Age-related alterations in MSCs have also been documented in several studies (98, 351, 352). For instance, MSCs derived from older individuals exhibit reduced superoxide dismutase activity, along with elevated concentrations of reactive nitrogen species (RNS) and reactive oxygen species (ROS) (353). As a result, MSCs suffer oxidative damage, which triggers apoptosis. Furthermore, aged MSCs have elevated expression of p53 and p21, which are known for their pro-apoptotic activities, and downregulated expression of the Notch1 receptor, which is linked to bone formation (353).

For therapies utilizing MSCs to achieve notable efficacy in addressing neurological disorders, it is imperative to consider the impact of these diseases on the regenerative capabilities of the cells. For instance, via downregulating pro-angiogenic factors, Diabetes Mellitus (type 2 diabetes) negatively affects MSC function and decreases their capacity to form new blood vessels (354). Furthermore, BM-MSCs obtained from diabetes patients show an increased tendency to grow into adipocytes and reduced paracrine secretion (355). Additionally, it has been noted that BMI affects adipocytes' capacity for differentiation and proliferation (356). Therefore, impaired DNA telomere length, cell proliferation, and differentiation are features of overweight persons. Together with this, cells have a reduced capacity for self-renewal and an early beginning of apoptosis. Furthermore, elevated BMI negatively impacts adipogenic and osteogenic differentiation in AT- and BM-MSCs, as demonstrated by significantly reduced cell division, increased senescence, and lessened adipogenic differentiation (357). It's noteworthy to notice that with a significant drop in weight, there is less damage to DNA and an improvement in both cell viability and replicative lifespan (358).

Lastly, pharmacological substances and treatment modalities such as immunosuppressive medications, anticancer medications, and radiation therapy also affect the characteristics of MSCs (359–361). Like this, long-term morphine usage reduces endothelial progenitor cell activation and angiogenesis (362). Furthermore, it hurts MSC differentiation and proliferation, changing their secretory capacities and impeding wound healing (362).

### 5.3 Heterogeneity

MSCs from diverse origins have dramatically variable features, even though several studies have demonstrated the intriguing advantages of MSCs in tissue regeneration, making them an appealing study topic in regenerative medicine (363). For instance, compared to cells isolated from adult tissues, MSCs derived from fetal tissues exhibit faster cell proliferation and the capacity to go through many *in vitro* passages before senescence (364). Conversely, adult-isolated BM-MSCs and AT-MSCs have a greater stemness, demonstrated by their capacity to form more fibroblast colonies (CFU-F) (365, 366). It's interesting to note that MSCs derived from specific donors may have distinct variations. Studies on BM-MSCs isolated from donors of various ages and sexes revealed notable variations in the cells' proliferation rates, osteogenesis, and activity levels of the marker for bone remodeling (alkaline phosphatase, or ALP) (367). It's interesting to note that there was no reported relationship between these and the donors' age or sex. However, other research has demonstrated that the age of the donor has a significant impact on the characteristics of BM-MSCs. For instance, cells taken from older adults showed reduced proliferation, higher apoptosis, and a lower ability to differentiate into osteoblasts (368). Heo et al. (366) also showed that MSCs generated from various tissues exhibit significant inter donor heterogeneity in distal-less homeobox 5 (DLX5) gene expression (366). To facilitate the identification of specific molecular and functional phenotypes associated with harvesting techniques and tissue sources, Colter et al. (369) categorized MSCs into three subpopulations based on their morphology: spindle-shaped proliferating cells resembling fibroblasts (Type I); large, flat cells characterized by prominent cytoskeletal structure containing numerous granules (Type II); and small, round cells exhibiting a high capacity for self-renewal (Type III) (369, 370).

### 5.4 Adverse effects

Further study is necessary to resolve the numerous issues and disagreements surrounding the use of MSCs in the human cell niche despite the positive results that MSC therapy offers. Thus, some of the primary possible risks associated with MSC therapy are as follows: (1) the possibility of pro-tumorigenic activity and undesirable cell type differentiation; (2) an uncontrolled immune response; (3) a brief survival period following implantation; (4) a lack of sufficient research on the differentiation capacities of MSCs; and (5) an inability to determine the best doses and mode of cell administration. By promoting tumor invasion by releasing CCL5 and preventing apoptosis by releasing pro-survival molecules like VEGF and bFGF, MSCs may demonstrate pro-tumorous activity (371–374). Therefore, introducing MSCs may cause an uncontrollable immunological response at the local or global level since they can explain both immunosuppressive and immunomodulatory effects (375). Regarding MSCs' brief lifetime after implantation, several studies have shown that, soon after transplantation, MSCs undergo massive mortality due to the activation of hypoxia signaling pathways and Caspase 3-mediated apoptosis. Remarkably, research by Deschepper et al. (376) revealed that ischemia circumstances (low pO2 and glucose depletion) caused the widespread mortality of MSCs at day 6. Still, hypoxic settings (low O2) allowed cells to survive until day 12 (376).

There are still several uncertainties about the differentiation ability and use of MSCs despite all the advantages of potential therapy approaches. These consist of their precise mode of action, safety during standard clinical usage, and tissue migration patterns (377). As a result, a large body of research indicates that various clinical indications and illnesses require distinct administration techniques for optimal therapeutic success (378, 379).

### 5.5 Tumor-promoting ability

Despite the ability of MSCs to migrate toward tumor locations, numerous studies caution against their pro-tumor effects, which include immunosuppression, stimulation of blood vessel formation, transformation into cancer-associated fibroblasts, prevention of cell death in cancer cells, enhancement of metastasis and tumor growth, initiation of epithelial-mesenchymal transition (EMT), and facilitation of resistance to drugs (334, 380–393). While the previously described research examined and measured the impact of local MSCs on tumor development and related activities, it is essential to consider MSCs' capacity to promote tumor growth while developing novel treatment strategies utilizing this cell population (394). MSCs play a crucial role in inhibiting the innate and adaptive immune responses. They achieve this by secreting several substances such as TGF α, TNF β, IFN γ, PGE2, NO, HLA-G, HGF, IL-1b, IL-1a, LPS, and IL-6 (375, 394). Consequently, these elements lessen the maturation of DC, the production of IgG, the activity of natural killer cells (NKCs), and the proliferation of effector T- and B-cells. The net effect is a decrease in anti-tumor immunity. Additionally, it has been demonstrated that MSCs stimulate tumor angiogenesis by releasing bFGF, VEGF, FGF-2, IL-8, IL-6, IGF-1, TNF, and TGF β. They also induce the development of new tumor arteries and change into smooth muscle cells and pericytes. MSCs can differentiate into cancer-associated fibroblasts (CAFs) and smooth muscle cells (56, 389, 394, 395).

### 5.6 Technical and societal challenges

The use of MSCs for therapeutic reasons necessitates highly competent specialists to prevent cell contamination and ease the deployment of a highly standardized technique. A consistent methodology detailing the proper processes for isolating and maintaining MSC cultures still does not exist, even though several clinical trials are now in progress (396). The rationale for the significance of this standardized protocol is its ability to simplify the process of comparing several experimental studies and clinical trials side by side to identify the best distribution strategy and concentration. The public's interest in stem cell treatment has grown, leading to a growth in biobanking in recent years. While these establishments offer their clients the ability to obtain versatile stem cells as needed, they are also vulnerable to potential exploitation (56, 396). This is especially noticeable in biobanks run by private companies, where there is a chance of privacy violations and health data being sold (397). Furthermore, users of these biobanking services seem to belong to a particular social group: well-educated, white, middle-class people. As a result, those who don't match these stereotypes—namely, those who are lower class, indigenous, or from culturally varied communities—are inadvertently left out (398). This prejudice not only prevents marginalized people from accessing biobanks but also has a detrimental effect on scientific research because biobank samples and data are used in many different types of studies. However, understanding also brings the power to address these problems with representativeness and inclusion, which we should actively work to address in the following years (56, 398).

## 6 Progress and prospects

Simultaneously, researchers have demonstrated that while stem cell therapy is utilized for treating brain damage, MSC therapy has potential disadvantages, such as the risk of tumor formation. However, this risk can be mitigated using exosomes derived from MSCs, which offer a safer alternative. Research has shown that MSCs can increase and decrease tumor growth and tumorigenesis in different conditions (Figures 1, 2). The tumor in its microenvironment tries not to be recognized by the immune system and creates a stable state by secreting inflammatory mediators. There is a lot of focus on the interaction between cancer cells, normal cells, and the matrix in the microenvironment because this interaction contributes to the milestones of cancer progression, such as angiogenesis, immune modulation, metastasis, and invasion, as well as resistance to apoptosis (399–401). Some studies have shown that MSCs migrate to the cancer microenvironment. This place supports the development of the tumor vascular system and influences immune reactions, thus modulating the tumor's response to antitumor therapy (Figure 2). MSCs have immunosuppressive solid properties that cause tumor cells to escape immune surveillance (341, 402–405). MSCs in the tumor microenvironment by pro-inflammatory cytokines IFN-γ, TNF-α, or IL-1β can be activated and secreted by macrophages and tumor cells (371, 406–408). IFN-γ produced by Th1 decreases, and IL-4 secretion increases through Th2, which minimizes antitumor immunity and immune response. Monocyte differentiation is controlled by IL-6 secreted by MSC toward DCs, and they reduce the ability of DCs to stimulate T cells (380, 409). Exosomes derived from MSCs have shown significant promise in the field of regenerative medicine, including the treatment of TBI. The three-dimensional culture of MSCs enhances the production of exosomes, thereby increasing their therapeutic efficacy. Exosomes offer inherent safety advantages over the administration of living cells. They reduce the risk of blockage in small vessels or irregular growth of transplanted cells. Unlike exogenous neural progenitor stem cell transplantation, MSC-derived exosomes stimulate endogenous neural progenitor stem cells to repair the damaged brain. The use of exosomes has several significant advantages, such as:

- There is no ethical problem with embryonic cells.
- They are less invasive.
- There is little or no tumorigenesis.

Exosomes are promising therapeutic agents because their complex cargo of protein and genetic material has different biochemical potential to participate in many biochemical and cellular processes, which is an essential feature in treating complex diseases with multiple secondary injury mechanisms such as TBI (394).

Future research should focus on optimizing MSC delivery methods and exploring the long-term effects of MSC therapy on neuroplasticity and cognitive function. Additionally, understanding the molecular mechanisms underlying MSC-mediated neuroprotection could pave the way for targeted therapies.

## 7 Conclusion

MSCs and their EVs represent innovative and promising therapeutic strategies for treating neurological disorders, particularly TBI, ischemic strokes, concussions, tumors, encephalitis, and brain hemorrhages. Through their multifaceted mechanisms, including immunomodulation, angiogenesis, neurogenesis, anti-inflammatory properties, and apoptosis regulation, MSCs and EVs address the complex pathophysiology of neurological injuries effectively. The key insights of the article highlight the ability of MSCs to target injured brain regions, thereby reducing neuroinflammation and promoting recovery. Similarly, cell-free therapeutic agents derived from MSC-derived EVs present enhanced safety profiles and circumvent challenges associated with using MSCs, such as immunogenicity and tumorigenicity. A review of preclinical and clinical evidence strongly suggests that MSCs and EVs are promising candidates for addressing unmet therapeutic needs in brain injuries due to their regenerative potential.

These advances, however, present several challenges that must be addressed. The sources of MSCs exhibit variability, recipient responses are heterogeneous, and concerns regarding immunocompatibility and potential tumorigenic risks warrant further investigation. Additionally, there is a need for standardization and thorough validation of optimal dosing, administration routes, and long-term safety profiles.
